# Supplementary material for: Learning curve and surgical outcome of robotic assisted colorectal surgery with ERAS program
Source: Sci Rep. 2022 Nov 29;12:20566. doi: 10.1038/s41598-022-24665-w (PMC9709162; doi:10.1038/s41598-022-24665-w)
Supplement: Supplementary file 2 — Supplementary Information 2. [file 41598_2022_24665_MOESM2_ESM.zip › RAL Raw data-Ñ[▒K/086/HS_ñw╝╨░O▒Kñσ.pdf]

正式摘要(電子簽章：000000218247028)		
W55 / W55 ☐ 英文標題(注意:無英文姓名-請補病人基本檔!)	☐【讀取】憑證	

臺中榮民總醫院

出院病歷摘要

轉入號： 轉出號：

[redacted]

(8)流水編號	
(9)入院日期	110/05/26 (2021) 直腸外科 W55 - 282
(10)轉科日期	
(11)出院日期	110/06/01 (2021) 住院天數計 6 日

診斷

- 入院 :colon cancer
- 出院 :Colon cancer, 35cm FAV, s/p Robot Assisted anterior resection on 2021/5/27

主訴

- colon cancer admitted for Robot Assisted Colectomy(L).

病史

- The 57-year-old female had no underlying disease.
  She suffered from LLQ pain and stool OB showed positive. Colonscope was arrnaged and showed colon cancer, 35CM FAV. Therefore, she was admitted for Robot Assisted Colectomy(L).

體檢發現

- 2021/05/26 住院前篩檢
  - General appearance :
    - easy looking , conscious clear , without cardiopulmonary distress
    - Vital signs自動帶入date 體溫(BT) 脈搏(P) 呼吸(R) 血壓(BP) 身高(BH) 體重(BW) BMI
      20210526 36℃ 74次/分 18次/分 134/78mmHg 156.8cm 56.5kg 23.0
  - HEENT :
    - Eyes: no anemic conjunctiva, no icteric sclera, normal EOM
    - ENT: no otorrhea, no rhinorrhea, no erythematous mucosa, no injected tonsil, no deformity
  - Neck: supple, freely movable, trachea midline,no deformity, no engorged jugular vein , no palpable thyroid or lymph node enlargement
  - Chest:
    - Inspection: Grossly normal, symmetric and fully expansion, no accessory muscle used, no operation scar, no spider angiomata, no gynecomastia
    - Palpation: no palpable mass or nodule, no subcutaneous emphysema, no tenderness
    - Ausculation: clear breathing sound
  - Heart:
    - Percussion: normal heart size
    - Ausculation: regular heart beat, normal S1, S2, no gallop, no murmur
  - Abdomen:
    - Inspection: in
      - flat contour, symetrically, no scar, no stria, no dilated veins, no rashes
    - Ausculation: normoactive bowel sound, no abdominal bruits
    - palpation: soft, no muscle guarding or rigidity, no local tenderness, no rebounding pain, no palpable mass, no palpable pulsation
    - Percussion: no shifting dulllness, no hepatomegaly, no splenomagaly
  -

Peripheral pulsation :	CA	BA	RA	FA	PA	PTA	DPA
Right side :	++	++	++	++	++	++	++/+++
Left side :	++	++	++	++	++	++	++/+++

- Genito-anus: no tenderness or palpable mass lesion or rectal shelf, normal external genitalia, normal anal tone, brown stool

CRS CONDITION

-
    - Lung

手術日期及方法

- 手術□期及方法20210527 Robot Assisted Colectomy(L)

住院治療經過

- After admission, pre op survey was OK an doperation was done smoothly. She tolerated the procedure well an doculd stand oral feeding gradually. Under relatively stable conditions, she will be discharged.

合併症

一般檢查

- 累積報告：

DATE	TIME	ALB	TP	BIL.T	BIL.D	ALKP	AST	ALT	LDH	AMY	LIPASE	NH3	Ferritin
1100527	1753	3.7		0.59	0.13	98	14	12					
1100531	0849	3.2		0.39				10					

DATE	TIME	WBC	RBC	HGB	HCT	MCV	PLT	BLST	NEUT	BAND	SEG	LYM	MON	EOS	BAS
1100527	1752	11940	3.69	11.6	36.4	98.6	304		92.5			6.4	0.8	0.1	0.2
1100531	0857	5510	3.38	10.9					77.7			16.5	3.8	1.6	0.4

DATE	TIME	NA	K	CL	BUN	CREAT	eGFR	CA	Mg	P	CRP	ESR	PCT	D-Dimer	LACTATE
1100527	1753	141	4.3	109	11	0.75	84.66	9.0							
1100531	0849	142	3.8		9	0.63	103.52	8.3							

DATE	TIME	GLUCOSE	HbA1C	CHOL	TG	HDL	LDL	KETONE	CK	CKMB	TRO-T	PT-P	APTT-P	NT-pro BNP
1100527	1753	121												

特殊檢查

病理報告

其它

出院時情況

- 3.改門診治療

出院指示

-

藥名	劑量	劑量單位	用法	頻次	數量	數量單位	現況	天數	單位
(□)Takepron OD tab 30mg	1	TAB	PO	QDAC	21	TAB	TH	21	天
Ultracet tab	1	TAB	PO	BID&HS	63	TAB	TH	21	天
Mag. Oxide tab	2	TAB	PO	TID	126	TAB	TH	21	天

出院計劃

主治醫師 2062F 蔣鋒帆 / 住院醫師 2309E 古朋育

正式摘要(**電子簽章：000000218247028)**

W55 / W55

doctorRank:B
